# 

**Published:** 1893-05-20

**Authors:** 


**OUR NEW OFFICES.**

428, Strand, W.C., will henceforth be the Office of this Journal.

**Notices of Births, Deaths, and Marriages,** are inserted in this column at a charge of 2s. 6d. for an announcement not exceeding four lines.

**DEATH**

In fondest remembrance of Jackie, adopted son of Jessie Brown, who passed away on Trinity Sunday, 6.15 p.m., 1891. The shadow of my little sunbeam is ever with me.—U.S.A.

### NOTICE TO CORRESPONDENTS.

All MS., Letters, Books for Review, and other matters intended for the Editor should be addressed The Editor, The Lodge, Porchester Square, London, W.

The Editor cannot undertake to return rejected M.S., even when accompanied by stamped directed envelope.

**Notice to Advertisers and Subscribers.**

All Advertisements, Orders for Copies of The Hospital, and Business Communications should be addressed to The Publisher, *not to the Editor*, The Hospital, 428, Strand, W.C.

The Cost of Subscription, post free, is as follows:—Per week, 2½d.; per quarter, 2s. 6d.; per half-year, 5s.; per annum, 8s. 6d. Foreign:—Per annum, 10s. 8d.; payable, in all cases, in advance.

**"Burdett's Hospital Annual," 1893.**

Fourth year of publication. 700 pp. Boards, gilt lettering. A most complete guide to British, Colonial, Indian, and American Hospitals, Dispensaries, Nursing and Convalescent Institutions, and Asylums. Price 5s. Published by The Scientific Press (Limited), 428, Strand, W.C.

**NOTICE.—The Index for Vol. XIII. (ending March 1893) is on sale at the Office of this Journal, price 2½d., post free.**

### SCRAPS AND GLEANINGS.

The famous laryngologists, Dr. Johamn Schnitzler, of Vienna, died recently, aged 58.

In the first week in May, 14 infants under one year old died in London from suffocation while in bed with their parents.

The Clothworkers' Company have promised to the Northern Polytechnic Institute a sum of £600 annually as an endowment.

The total sums collected in private boxes for the Victoria Hospital, Lewis, on Hospital Sunday, was £78. In all over £200 was raised.

£2,191,172 was the sum spent on the relief of paupers by the various relief boards in England and Wales during the last six months of the year 1892.

There are now 20 medical officers of health receiving certain salaries from the County Council of London; whilst the total number of medical officers of health in the metropolis is 53.

As a reaction against professional fasting, a man at Paris has recently gone to the other extreme, and as a professional feat, has taken eighty glasses of beer in an evening at the Moulin Rouge.

The Early Closing Association has an auxiliary in a league lately established at Stoke Newington, and calling itself by the extravagantly lucid title of "The Ladies' Shop Before Two on Thursdays League."

The average of patients admitted monthly to the Horton Infirmaay at Banbury is stated to be 22. The books of the Provident Dispensary for the year give the number of 886 patients, the receipts amounting to £141.

Milk has been proved by a French physician to be an excellent dressing for burns. Compresses soaked through with milk are laid on the part affected twice a day, and the result is declared highly beneficial to the sufferer.

A corporation has lately been organised in New York with the avowed intention of "Performing the clerical, financial, and legal work necessary to the proper conduct and protection of the business affairs of medical men."

### BOOKS RECEIVED.

WOMEN'S EMANCIPATION UNION.

"Woman Free." By Ellis Ethelmer.

SIMPKIN, MARSHALL, AND CO.

"Recreation." By William Odell, F.R.C.S.
"The Germ-Plasm: A Theory of Heredity." By August Weismann. Translated by W. Newton Parker and Harriet Rönnfeldt.

JOHN MURRAY.

"Health Hints for Central Africa." By Horace Waller.

SPRAGUE AND CO.

"On the Application of Suitable Mechanism to a Case of Amputation of Both Hands." By F. Gustav Ernst.

BAILLIERE, TINDALE, AND CO.

"Notes on Medicinal Remedies." By J. B. Stephenson.

"Aids to Biology." By Joseph W. Williams.

"The Hygiene of Beauty." By Dr. E. Monin (Paris).

H. K. LEWIS.

"Accidents and Emergencies." By Charles W. Dulles, M.D. (4th Edition.)

H. ALABASTER AND CO., LONDON.

"Domestic Electric Lighting Treated from the Consumer's Point of View." By E. C. de Segundo.

THE RELIGIOUS TRACT SOCIETY.

"Modern Scepticism compared with Christian Faith." By Rev. M. Kaufmann.

**Periodicals and Pamphlets.**—London, The Review of Reviews, The Religious Review of Reviews, The Review of the Churches, The Quarterly Therapeutic Review, The St. James's Budget. The Clinical Journal, The Bristol Medico-Chirurgical Journal, The Cornhill Magazine, The Indian Medico Chirurgical Review, The Australian Agriculturist, The Glasgow Medical Journal, Spiritualistic Experiences of W. Jenner Champernowne, Notes upon an Outbreak of an unusual form of Skin Disease at the Greenock Parochial Asylum and Poor-house, A Guide to Rome, by Rev. S. Lunn, 5, Ensleigh Gardens, W. The Religious Tract Society: The Leisure Hour, The Sunday at Home, The Girl's Own Paper, The Boy's Own Paper, Light in the Home, Child's Companion, Our Little Dots, The Cottager and Artisan, Friendly Greetings.

### MEDICAL VACANCIES AND APPOINTMENTS.

### APPOINTMENTS.

G. W. Ancrum, M.B., C.M., L.R.C.S., L.R.C.P.Edin., appointed House Surgeon to the General Infirmary at Gloucester and the Gloucestershire Eye Institution. H. G. Barling, M.B.Lond., F.R.C.S.Eng., appointed Professor of Surgery at Mason College, Birmingham. M. Beck, M.S., M.B.Lond., appointed Examiner in Surgery at the University of London. D. T. Belding, L.R.C.P. Lond., M.R.C.S.Eng., appointed Medical Officer for the East Dereham District of the Mitford and Launditch Union. A. Bicknell, M.B., Ch.B.Vict., appointed Medical Officer for the Brownlow Hill Workhouse of the Parish of Liverpool. A. B. Brockway, M.R.C.S., L.R.C.P., appointed Resident Medical Officer to the Brisbane General Hospital. G. H. Browne, L.R.C.P.Edin., L.F.P.S.Glas., reappointed Medical Officer of Health for the Brynmawr Urban Sanitary District. J. Cavafy, M.D.Lond., appointed Examiner in the Practice of Medicine at the University of London. C. M. Chapman, M.B., C.M.Edin., appointed House Physician to the Bradford Infirmary. C. Christopherson, L.R.C.P.Lond., M.R.C.S.Eng., appointed Assistant Surgeon to the White Rock Hospital, Hastings. W. Clegg, M.R.C.S.Eng., L.S.A., reappointed Medical Officer of Health for the Boston Urban Sanitary District. W. Clibborn, M.D.Dub., L.R.C.S.I., reappointed Medical Officer of Health for the Bridport Urban Sanitary District. C. J. Cullingworth, M.D., appointed Examiner in Obstetric Medicine at the University of London. D. J. Cunningham, M.D., D.Sc., F.R.S., appointed Examiner in Anatomy at the University of London. J. W. Davies, M.B., C.M.Edin., M.R.C.S.Eng. reappointed Medical Officer of Health for the Ebbw Vale Urban Sanitary District. C. Dawson, M.D.Durh., L.R.C.P., M.R.C.S., reappointed Medical Officer of Health for Rawdon. W. H. Dawson, M.D.Durh., M.R.C.S., appointed Medical Officer of Health for the Malvern Urban Sanitary District of the Upton-upon-Severn Union. C. J. Evers, M.D.Durh., M.R.C.S., reappointed Medical Officer of Health for Faversham Borough and Port Sanitary Authority. H Fenton, appointed Physician to the Chelsea Hospital for Women. H. Finley, M.B.Lond., M.R.C.S.Eng., L.R.C.P.Lond., appointed Junior House Surgeon to the Cumberland Infirmary, Carlisle.

### VACANCIES.

**The following vacancies are announced this week, the amount of salary in each case being given after the name of the institution:**

### Resident Medical Officers.

Cancer Hospital (Free), Fulham Road, S.W. House Surgeon. Appointment for six months. Salary at the rate of £50 per annum, with board and residence. Applications to the Secretary by May 22nd.

Chorlton-upon-Medlock Dispensary, Manchester. Resident House Surgeon, doubly qualified, unmarried. Salary £100 per annum, with furnished rooms and attendance. Applications to the Honorary Secretary by May 25th.

Leicester Infirmary. Assistant House-Surgeon. Salary £80 per annum, with board, apartments, and washing. Applications to the Secretary, 24, Friar Lane, Leicester, by May 27th.

New Hospital for Women, 144, Euston Road, N.W. Resident Medical Officer. Three Clinical Assistants. Applications to the Secretary by May 26th.

St. Luke's Hospital, E.C. Clinical Assistant. Appointment for six months. Board and residence provided. Applications to Percy de Bathe, M.A., Secretary, by May 25th.

### Non-Resident Medical Officers.

Hendon Union, Edgware. Medical Officer for the Willesden No. 2 District. Salary £85 per annum, and extra fees for surgical operations and midwifery. Applications to the Clerk, at the Union Offices, by May 22nd.

National Dental Hospital, Great Portland Street, W. Assistant Dental Surgeon; Anæsthetist. Applications to the Secretary by May 15th.

North Dublin Union, Coolock and Drumcondra Dispensary. Medical Officer. Salary £46 11s. 8d. per annum, with £5 yearly as Medical Officer of Health, registration and vaccination fees. Applications to Mr. Jno. Doyle, Honorary Secretary, Moyne Lodge, Portmarnock. Election on May 22nd.

North-West London Hospital, Kentish Town Road, N.W. Assistant Physician. Applications to Alfred Craske, Secretary, by May 26th.

Royal Infirmary, Bristol. Honorary Assistant Physician. Applications and testimonials to J. F. Shekleton, Secretary and House Governor (who can forward a list of the Committee of Election) by June 10th.

St. Pancras and Northern Dispensary, 126, Euston Road. Surgeon. Applications to H. Peter Bodkin, Honorary Secretary, 23, Gordon Street, Gordon Square, W.C., by May 25th.

Sedbergh Union, Sedbergh, Yorkshire. Medical Officer and Public Vaccinator of the parish of Dent. Salary £50, and fees for midwifery cases, surgical operations, and vaccination. Applications to W. Robinson, Clerk to the Guardians, by May 23rd.

### MEDICAL APPOINTMENTS.

**LEICESTER INFIRMARY.**

**NORTH-WEST LONDON HOSPITAL,**
Kentish Town Road.

**ROYAL INFIRMARY, BRISTOL.**

